# 691. Association between *Clostridioides difficile* Colonization, Rectal Swab NAAT Positivity, and Environmental Contamination

**DOI:** 10.1093/ofid/ofad500.753

**Published:** 2023-11-27

**Authors:** Mary Morgan Lee, Tiffany Hink, Erin Newcomer, Skye Fishbein, Kimberly Reske, Emily Struttmann, Zainab Iqbal, Candice Cass, Jennie H Kwon, Margaret A Olsen, Carey-Ann Burnham, Gautam Dantas, Erik R Dubberke

**Affiliations:** Division of Infectious Diseases, Washington University School of Medicine, St. Louis, Missouri, USA, St. Louis, Missouri; Washington University, St. Louis, Missouri; Division of Laboratory & Genomic Medicine, St. Louis, Missouri, USA, St. Louis, Missouri; Division of Laboratory & Genomic Medicine, St. Louis, Missouri, USA, St. Louis, Missouri; Washington University, St. Louis, Missouri; Division of Infectious Diseases, Washington University School of Medicine, St. Louis, Missouri, USA, St. Louis, Missouri; Division of Infectious Diseases, Washington University School of Medicine, St. Louis, Missouri, USA, St. Louis, Missouri; Washington University, St. Louis, Missouri; Washington University - School of Medicine, St. Louis, MO; Washington University School of Medicine in St. Louis, St. Louis, Missouri; Washington University, St. Louis, Missouri; Washington University School of Medicine in St Louis, St. Louis, MO; Washington University, St. Louis, Missouri

## Abstract

**Background:**

*Clostridioides difficile* (CD) colonization is common among hospitalized patients. Screening for toxigenic CD (TCD) colonization with rectal swab nucleic acid amplification tests (NAAT) may identify people at risk for CDI and help prevent onward TCD transmission, especially in immunocompromised patients with long hospitalizations. The study objective was to test whether rectal swab NAAT could detect TCD colonized patients and whether NAAT results identify patients that contribute to environmental contamination.

**Methods:**

This was a prospective cohort study on two BMT/Leukemia units. Stool specimens, rectal swabs, and environmental swabs (bedrail, room sink, keyboard) were collected from patients at admission and weekly and cultured semi-quantitatively for TCD. NAAT (Cepheid) was performed on rectal swabs (eSwabs; Copan Diagnostics). The relationship between stool TCD concentration, NAAT Ct, and a positive environmental culture was evaluated using univariate logistic regression. Bleach was used for daily and terminal discharge cleaning of all rooms. Toxigenicity was determined by detection of *tcdA* or *tcdB* by whole genome sequencing (WGS).

**Results:**

From January 2019 - July 2019, 659 stool specimens and 495 rectal swabs were collected from 384 patients. Specimens from 554 patient-days were cultured and 126 (23%) were positive. Of those, 88 (70%) were TCD. 21 of 987 (2%) environmental swabs were TCD positive; 18 (86%) were from bedrails. Among 61 unique patient calendar days with paired rectal swab NAAT and stool culture results (paired samples), overall sensitivity of rectal swab NAAT to detect toxigenic CD colonization compared to culture was 44%, with 4/16 (25%) of people colonized with > 0 - < 10,000 CFU/g of TCD and 7/9 (78%) with ≥ 10,000 CFU/g TCD (Figure 1). There was no relationship between stool culture or rectal swab NAAT result and risk of a positive environmental swab (Figure 2).

**Figure 1.**
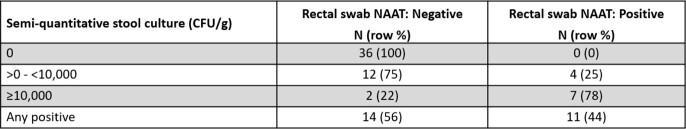
Toxigenic C. difficile semi-quantitative culture and rectal swab NAAT on paired patient-day of specimen collection (N=61 patient-days)

**Figure 2.**
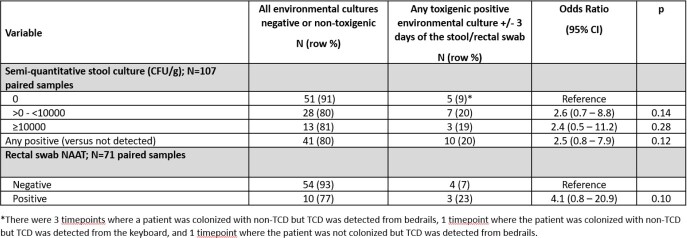
Toxigenic C. difficile semi-quantitative stool and rectal swab NAAT results and environmental contamination by paired patient-day of specimen collection

**Conclusion:**

Rectal swab NAAT may not be sufficiently sensitive to screen for TCD colonization that contributes to environmental contamination.

**Disclosures:**

**Erik R. Dubberke, MD, MSPH**, Abbott: Advisor/Consultant|AstraZeneca: Advisor/Consultant|Ferring Pharmaceuticals: Advisor/Consultant|Ferring Pharmaceuticals: Grant/Research Support|Merck and Co.: Advisor/Consultant|Pfizer: Advisor/Consultant|Pfizer: Grant/Research Support|Seres Therapeutics: Advisor/Consultant|Summit: Advisor/Consultant|Theriva Biologics: Grant/Research Support

